# Immunization Elicits Antigen-Specific Antibody Sequestration in Dorsal Root Ganglia Sensory Neurons

**DOI:** 10.3389/fimmu.2018.00638

**Published:** 2018-04-16

**Authors:** Manojkumar Gunasekaran, Prodyot K. Chatterjee, Andrew Shih, Gavin H. Imperato, Meghan Addorisio, Gopal Kumar, Annette Lee, John F. Graf, Dan Meyer, Michael Marino, Christopher Puleo, Jeffrey Ashe, Maureen A. Cox, Tak W. Mak, Chad Bouton, Barbara Sherry, Betty Diamond, Ulf Andersson, Thomas R. Coleman, Christine N. Metz, Kevin J. Tracey, Sangeeta S. Chavan

**Affiliations:** ^1^Center for Biomedical Science, Feinstein Institute for Medical Research, Northwell Health, Manhasset, NY, United States; ^2^Center for Genomics and Human Genetics, Feinstein Institute for Medical Research, Northwell Health, Manhasset, NY, United States; ^3^Elmezzi Graduate School, Feinstein Institute for Medical Research, Northwell Health, Manhasset, NY, United States; ^4^Donald and Barbara Zucker School of Medicine at Hofstra/Northwell, Hempstead, NY, United States; ^5^GE Global Research Center, Niskayuna, NY, United States; ^6^The Campbell Family Institute for Breast Cancer Research, University Health Network, Toronto, ON, Canada; ^7^Center for Bioelectronic Medicine, Feinstein Institute for Medical Research, Northwell Health, Manhasset, NY, United States; ^8^Center for Immunology and Inflammation, Feinstein Institute for Medical Research, Northwell Health, Manhasset, NY, United States; ^9^Center for Autoimmune, Musculoskeletal and Hematopoietic Diseases, Feinstein Institute for Medical Research, Northwell Health, Manhasset, NY, United States; ^10^Department of Women’s and Children’s Health, Karolinska Institutet, Solna, Sweden; ^11^Center for Molecular Innovation, Feinstein Institute for Medical Research, Northwell Health, Manhasset, NY, United States

**Keywords:** DRG, sensory neurons, antibodies, neural circuits, inflammation

## Abstract

The immune and nervous systems are two major organ systems responsible for host defense and memory. Both systems achieve memory and learning that can be retained, retrieved, and utilized for decades. Here, we report the surprising discovery that peripheral sensory neurons of the dorsal root ganglia (DRGs) of immunized mice contain antigen-specific antibodies. Using a combination of rigorous molecular genetic analyses, transgenic mice, and adoptive transfer experiments, we demonstrate that DRGs do not synthesize these antigen-specific antibodies, but rather sequester primarily IgG_1_ subtype antibodies. As revealed by RNA-seq and targeted quantitative PCR (qPCR), dorsal root ganglion (DRG) sensory neurons harvested from either naïve or immunized mice lack enzymes (i.e., RAG1, RAG2, AID, or UNG) required for generating antibody diversity and, therefore, cannot make antibodies. Additionally, transgenic mice that express a reporter fluorescent protein under the control of Igγ1 constant region fail to express *Ighg1* transcripts in DRG sensory neurons. Furthermore, neural sequestration of antibodies occurs in mice rendered deficient in neuronal *Rag2*, but antibody sequestration is not observed in DRG sensory neurons isolated from mice that lack mature B cells [e.g., *Rag1* knock out (KO) or μMT mice]. Finally, adoptive transfer of *Rag1*-deficient bone marrow (BM) into wild-type (WT) mice or WT BM into *Rag1* KO mice revealed that antibody sequestration was observed in DRG sensory neurons of chimeric mice with WT BM but not with *Rag1*-deficient BM. Together, these results indicate that DRG sensory neurons sequester and retain antigen-specific antibodies released by antibody-secreting plasma cells. Coupling this work with previous studies implicating DRG sensory neurons in regulating antigen trafficking during immunization raises the interesting possibility that the nervous system collaborates with the immune system to regulate antigen-mediated responses.

## Introduction

The mammalian immune system has acquired capacity to recall prior exposure to a vast number of potential antigens during the lifetime of the host. The development of this long-lasting immunity begins in a lymph node as an acute encounter between a naïve T cell and an antigen presenting cell. T and B lymphocytes recirculate between the lymphatic fluid, lymph node, and blood, entering the lymph node through high endothelial venules. Once inside the node, lymphocytes migrate through the T-cell zone and B-cell follicles until encountering an antigen presented by CD169+ macrophages or other antigen-presenting dendritic cells (1). The temporal and spatial control of antigen transport into the lymph node is dependent upon both the flow of isolated antigen *via* afferent lymphatic channels, and the trafficking into lymph nodes of intracellular antigen in antigen-presenting cells (APCs). Significant changes in lymph node architecture, inhibition of lymphocyte egress, and expansion of the lymph node stroma occur following antigen-mediated activation of intranodal immune responses.

We recently discovered that neural signals provide an essential mechanism in lymph nodes to retain antigen and lymphocytes (2). Lymph nodes are innervated by both sensory (afferent) and motor (efferent) neurons, with abundant innervation of the APCs in the T-cell zone, subsinoidal layer, and cortical extrafollicular zones (3, 4). The neuronal density within the lymph nodes is dynamic, expanding significantly following antigenic stimulation of an early lymphocyte response (5, 6). In naïve mice exposed to the antigen keyhole limpet hemocyanin (KLH) in the hind paw for the first time, we observed that KLH flowed rapidly from the popliteal lymph node (adjacent to the injection site) to the sciatic lymph node by traveling up the lymphatic chain. Surprisingly, prior exposure to KLH significantly impaired the flow of KLH from the popliteal to the sciatic lymph node during subsequent antigen exposure. This restriction of antigen flow from distal to proximal lymph nodes was antigen-specific and required functioning sensory neural signals, because inhibiting neural signals to the lymph node region restored antigen flow in immunized animals (2). The neural mechanism that restricted antigen flow is mediated by a subset of Na_V_1.8+ sensory neurons, which include nociceptors, because antigen failed to accumulate in the distal lymph node of animals rendered deficient in Na_V_1.8+ neurons (2). Early work had previously established that immune complexes can interact *via* Fc receptors expressed on sensory neurons to induce intracellular signaling mechanisms that require transient receptor potential canonical 3 (TRPC3) channel and the Syk-PLC-IP3 pathway (7, 8) that mediates the release of calcitonin gene-related peptide (CGRP) and substance P (9). Immunohistochemistry using labeled antigen revealed colocalization of labeled antigen and FcγRI receptors in neurons at the site of antigen injection in the paw (2). Thus together, these studies implicate sensory neurons in regulating antigen trafficking during immunization through a pathway that requires Na_V_1.8 and FcγR.

As we continued to explore the role of sensory neurons in immunization, we were very surprised to observe that sensory neurons obtained from the dorsal root ganglia (DRGs) of immunized animals contained abundant levels of antigen-specific antibodies. While others have reported the localization of antibodies to neurons in brain, to our knowledge, the localization of antibodies to sensory neurons in peripheral tissues has not been previously described. *Rag2* expression has been identified in brain neurons, but convincing evidence that neurons can synthesize antigen-specific antibodies is lacking. Accordingly, here we used molecular genetic analyses, and transgenic and chimeric mice to show for the first time that dorsal root ganglion (DRG) sensory neurons in immunized animals accumulate lymphocyte-derived, antigen-specific IgG_1_.

## Materials and Methods

### Animals

Six- to eight-week old male C57BL/6J (CD45.2), congenic B6.SJL-Ptprc^a^*Pepc*^b^/BoyJ (also known as CD45.1;B6 Cd45.1, Pep Boy; or Ly5.1), B6.129S7-*Rag1*^tm1Mom^/J [also known as *Rag1* knock out (KO)]; B6.129P2(Cg)-*Ighg1^*tm1(cre)Cgn*^*/J (Cγ1-cre), B6.Cg-*Gt(ROSA)26Sor^*tm14(CAG-tdTomato)Hze*^*/J (Ai14(RCL-tdT)-D), C57BL/6-*Rag2^tm1Cgn^*/J (Rag2 flox), and B6.Cg-Tg(Syn1-cre)671Jxm/J and B6.129S2-*Ighmtm1Cgn*/J (μMT) mice were purchased from the Jackson Laboratory (Bar Harbor, ME, USA). Syn1-cre mice were crossed to *Rag2*^flox/flox^ mice to generate Syn-cre/Rag2^flox/+^. Syn1-cre/Rag2^flox/+^ mice were backcrossed to Rag2^flox/flox^ mice to generate Syn1-cre/Rag2^flox/flox^ (Rag2^KO^) mice. To generate Ighg1-tdTomato mice, B6.129P2(Cg)-*Ighg1^*tm1(cre)Cgn*^*/J mice were bred with tdTomato reporter mice (B6.Cg-*Gt(ROSA)26Sor^*tm14(CAG-tdTomato)Hze*^*/J). Mice were housed in the animal facility of the Feinstein Institute for Medical Research under standard temperature and 12 h light and dark cycles. All mice were allowed to acclimate for at least 7 days before experimentation. All protocols used in this study involving animals were approved by the Institutional Animal Care and Use Committee (IACUC) of the Feinstein Institute for Medical Research, and all experimentation was in accordance with the National Institutes of Health guidelines for animal care (Guide for the Care and Use of Laboratory Animals, National Research Council 2011).

### Generation of Chimeric Mice

The recipient mice were sub-lethally irradiated with two split doses (WT: 5 Gy; *Rag1* KO: 4 Gy) delivered 14 h apart, and their immune systems were rescued *via* BM transplantations from *Rag1* KO (CD45.2) or WT (CD45.1) mice, respectively. Antibiotic gel diet (MediGel^®^ TMS) was given to animals before and after irradiation. Donor mice were euthanized by CO_2_ asphyxiation/cervical dislocation, and the femur and tibia were harvested. BM cells were collected by gently flushing the femurs and tibia and suspended at a concentration of 2 × 10^6^ cells in 200 µL of sterile Hank’s balanced salt solution (HBSS). Cells were administered intravenously (retro-orbital injection) to recipient mice 4–5 h after the second dose of irradiation. Reconstitution of the BM in recipient mice was confirmed after 2–4 weeks by assessing numbers of CD45.1 or CD45.2-positive cells in the peripheral blood by flow cytometry (reconstitution was >98%).

### Immunization Protocols

For all immunohistochemical staining of DRGs, RNA-seq, and ELISpot assays, mice were injected intraperitoneally with either 50% alum (Imject™ Alum; Thermo Fisher Scientific, Waltham, MA, USA) or 50% alum + 100 µg KLH (Calbiochem, San Diego, CA, USA) or 50% alum + 100 µg ovalbumin (OVA; Invivogen, San Diego, CA, USA) in 200 µl of 0.9% saline on day 0 and day 14. DRGs were harvested 14 days later (see below). For *in vivo* analysis of gene expression by quantitative PCR (qPCR), mice were injected with either 50% alum or 50% alum + 100 µg KLH in 200 µl of 0.9% saline on days 0, 14, and 28, and DRGs were collected 24 h post each injection.

### Collection of Whole DRGs and Isolation of Sensory Neuron Preparations

Dorsal root ganglia from thoracic and lumbar regions were harvested from mice immediately following euthanasia by CO_2_ asphyxiation. DRGs from the thoracic and lumbar regions were chosen because they have been shown to innervate the peritoneal cavity (10). DRGs were gently collected from the cavities along the lateral vertebral column as described previously (11) and enriched for neuronal populations, as described by Ref. (12). For the isolation of sensory neurons, DRGs were digested with 1 µg/ml collagenase/dispase (Roche Life Science, Germany) in neurobasal medium (Gibco, Thermo Fisher Scientific, Waltham, MA, USA) for 1 h at 37°C on a rotator-shaker. Following digestion, DRGs were washed with HBSS (Gibco), triturated using fire-polished glass Pasteur pipettes (Fisher Scientific, Waltham, MA), and filtered through a 70-µm strainer. The cell suspension was layered onto 15% BSA in HBSS and centrifuged at ×129*g* for 20 min without brake. The cell pellet was re-suspended in neurobasal medium. B lymphocytes were depleted from cell preparations using Dynabeads™ mouse pan B purification kit (Invitrogen, USA), and cell purity was assessed by flow cytometry. The resulting sensory neuron preparation was resuspended in complete neurobasal medium [neurobasal™ medium supplemented with penicillin-streptomycin (Gibco), GlutaMax™ (Gibco), B-27^®^ serum-free supplement (Gibco), and 50 ng/ml NGF (Sigma-Aldrich)].

### Immunohistochemical Staining

Whole mount DRGs were harvested from mice immediately after euthanasia by CO_2_ asphyxiation as described above and fixed with 4% PFA for 2 h. Fixed specimens were incubated overnight in 30% sucrose/PBS, washed, embedded, and frozen in optimal cutting temperature compound (Tissue-TEK OCT, Electron Microscopy Sciences, VWR, Radnor, PA, USA). 14 µm thick frozen sections were mounted on the Superfrost Plus glass slides (Thermo Fisher Scientific). The tissue sections were blocked and permeabilized with blocking solution containing rabbit serum and 0.1% Triton X-100. For immunostaining, blocked/permeabilized sections were stained with AlexaFluor 488-rabbit F(ab′)_2_ anti-mouse IgG (H + L) (1:1,000; Abcam, San Francisco, CA, USA), AlexaFluor 647-rabbit anti-NeuN (1:50; Abcam), and DAPI. Sections were mounted in ProLong anti-fade mounting medium (Thermo Fisher Scientific) and imaged using an LSM880 laser scanning confocal microscope (Carl Zeiss) using X40 Zeiss plan-apochromatic oil objective with Z stacks. The numbers of NeuN-positive and NeuN-positive IgG-positive neurons were quantified in five DRG sections pooled from three animals using ImageJ. The percentages of IgG-positive neurons were quantified by counting the number of neurons double positive for IgG and NeuN divided by the total NeuN-positive neurons in each DRG section and then multiplied by 100.

### Primary Cultures of Isolated DRG Sensory Neuron Preparations

Dorsal root ganglion neuron preparations were cultured in complete neurobasal medium [neurobasal™ medium supplemented with penicillin-streptomycin (Gibco), GlutaMax™ (Gibco), B-27^®^ serum-free supplement (Gibco), 50 ng/ml NGF (Sigma-Aldrich), 10% fetal bovine serum, and 1× β-mercaptoethanol] for DRG neuron activation assays (see below).

### ELISpot Assay

Dorsal root ganglion sensory neurons (isolated and prepared as described above) were cultured on anti-IgG or OVA- or KLH-coated ELISPOT plates and developed using the standardized ELISpot kit (Mabtech Inc., Cincinnati, OH, USA). Briefly, 96 well flat bottom multi-screen filter plates (Millipore, Billerica, MA, USA) were coated with 100 µL per well of KLH (50 µg/ml) or OVA (50 µg/ml) or anti-IgG antibody (15 µg/ml). The plates were incubated overnight at 4°C, washed with PBS, and blocked using complete neurobasal medium. DRG sensory neurons (isolated and prepared as described above from five mice per group for eight wells) suspended in complete neurobasal medium were cultured in the plates overnight at 37°C with 5% CO_2_. ELISpots were developed as per the manufacturer’s protocol and scanned and analyzed at Cellular Technology Ltd. (Shaker Heights, OH, USA).

### FluoroSpot Assay

Dorsal root ganglion sensory neurons (isolated and prepared as described above) were cultured in anti-mouse IgG-coated plates and incubated overnight at 37°C with 5% CO_2_. FluoroSpots were developed using the fluorescent tagged mouse IgG_1_, IgG_2a_, and IgG_3_ detection kit as per the manufacturer’s protocol (Cellular Technology Ltd.) and scanned and analyzed at Cellular Technology Ltd.

### *In Vitro* B Cell and DRG Neuron Activation Studies

Splenic B cells were purified from 7- to 10-week-old naïve C57BL/6 mice using the EasySep B Cell Isolation Kit (STEMCELL Technologies, Cambridge, MA, USA). B cell purity (CD19-positive cells) was confirmed (>99%) using flow cytometry. Isolated B cells were cultured in complete DMEM containing penicillin, streptomycin, glutamine, β-mercaptoethanol (1× each) and 10% fetal bovine serum at 3 × 10^6^ cells/ml per well (24 well plate) in duplicate. DRG neurons were isolated from naïve C57BL/6 mice and depleted of B cells, as described above. DRG neurons from five naïve mice were cultured in 0.25 ml per well (48 well plate) in duplicate per condition in complete neurobasal media (described above) containing 10% fetal bovine serum and 1× β-mercaptoethanol. B cells and DRG neurons were stimulated with (1) vehicle (untreated cells); (2) LPS (25 µg/ml) + IL-4 (10 ng/ml); and (3) anti-CD40 (10 µg/ml) + IL-4 (10 ng/ml). B cells were harvested 24, 48, and 72 h post stimulation, and DRG neurons were harvested 48, 72, 96, and 120 h post stimulation, and markers of antibody production and the generation of plasmablasts/plasma cells were assessed by qPCR (e.g., *Rag2, Aicda*, and *Prdm-1*, as described below; see Table 1 for primers).

**Table 1:** Quantitative PCR (qPCR) primers and probes.

Gene name	Forward sequence	Reverse sequence	Probe
*Rag1*	aggcctgtggagcaaggta	gctcagggtagacggcaag	46
*Rag2*	tgaataaagatgtcaacagccaat	ggtaccctcaatccccactt	29
*Aicda*	tcctgctcactggacttcg	gcgtaggaacaacaattccac	71
*Ungv1v2*	ccatggggatttgtcagg	acagtgaggacggcgttg	101
*Cd19*	aaggtcattgcaaggtcagc	ctgggactatccatccacca	21
*Cd138*	gagggctctggagaacaaga	tgtggctccttcgtccac	5
*Prdm1*	cgctatgactttggtgcttg	accctcacctctgcactga	108
*Hprt1*	tcctcctcagaccgctttt	cctggttcatcatcgctaatc	95

*Forward and reverse primers and probes for indicated mouse genes (Roche Universal ProbeLibrary); v1v2 = variant 1 variant 2*.

### RNA Isolation

Naïve mouse spleens (positive control) and DRG neuron preparations isolated from naïve, alum-injected, and alum + KLH-immunized mice (*n* = 10 mice per condition, pooled) were lysed with Qiazol lysis reagent (Qiagen, Germantown, MD, USA) and stored at −80°C until RNA preparation (DRG neurons from 10 mice yielded approximately 50 µg RNA). Spleen and DRG RNA were isolated using the RNeasy Universal Kit (Qiagen). For the *in vitro* activation assays (described above), RNA was isolated from DRG neurons using the RNeasy Universal Kit (Qiagen) and from B cells using the RNeasy Mini Kit (Qiagen). For RNA-seq studies, RNA quality was analyzed using the Bioanalyzer (Agilent Technologies, Santa Clara, CA, USA); RNA integrity numbers (or RIN values) were approximately 8.0. All RNA samples had OD260:OD280 ratios >1.8.

### RNA-Seq and Analysis

The sequencing mRNA libraries (for paired-end reads) were prepared using RNA isolated from DRG neurons purified from either alum-injected or alum + KLH-immunized C57BL/6J mice (*n* = 10 mice per group, pooled) using the TruSeq Stranded mRNA Sample Preparation Kit (Illumina, San Diego, CA, USA), according to the manufacturer’s instructions. As an exploratory step for characterizing the DRG neuron preparations, deep RNA sequencing was performed on the Illumina NextSeq 500 platform. The read depth was at least 60 M reads per sample using 75 base pair paired-end reads for a total output of ~18 Gb. The raw image files from the NextSeq sequencer were de-multiplexed and converted to fastq files using the bcl2fastq BaseSpace App (Illumina). RNA-seq analysis was performed on the existing annotated reference transcriptome (mouse) – no alternate transcript discovery was performed. Adapter sequences and low-quality base reads were trimmed from fastq files using cutadapt. The fastq files were aligned to the iGenome mm10 reference sequence from Illumina, and gene expression was quantified using kallisto.^1^ Data are reported as transcripts per million (TPM), which are considered more comparable between samples of different origins and composition, and the most frequently reported RNA-seq gene expression values (13). An expression value >3.0 TPM (transcripts per million) was used as a threshold for detectable expression. The RNA-seq data discussed here have been deposited in the NCBI Gene Expression Omnibus (GEO) (14) and are accessible through GEO Series accession number GSE108428.^2^

### RNA-Seq Deconvolution Analysis

We utilized DeconRNASeq software (15) to perform RNA-seq deconvolution analysis to infer cell type compositions of the neuronal preparations isolated from alum-injected and alum + KLH-immunized mice. DeconRNASeq, a freely available software package written in R, was downloaded from http://bioconductor.org/packages. DeconRNASeq uses a globally optimized non-negative decomposition algorithm for estimating the relative proportions of distinctive cell types in a mixture sample. High-quality pure cell-type signatures were derived from RNA sequencing data on single cells from the mouse lumbar DRGs (16). The data are available from the GEO (GSE59739), and the normalized data expressed as reads per million (RPM) were downloaded from http://linnarssonlab.org/drg/. This dataset contains single cell RNA-seq measures of 799 cells as well as clustering assignments of 731 of these cells by principal component analysis. The five principal cell classes included 109 non-neuronal (NoN) cells, 81 peptidergic nociceptor (PEP) cells, 169 non-pep (NP) cells, 139 neurofilament containing (NF) cells, and 233 tyrosine hydroxylases containing (TH) cells. We averaged the RNA-seq measures for each of the five cell classes. We excluded genes in which the mean RPKM (reads per kilobase of TPM mapped reads) value for the cells types was below a 0.1 cutoff value. There were 11,776 genes that passed this constraint and were used in the deconvolution analysis. Using the deconvolution analysis, we analyzed relative proportions of distinctive cell types in our groups. Based on pure cell-type signatures, approximately 73% of our DRG neuron preparations were neuronal (Figure S1 in Supplementary Material). Of the cells with a neuronal origin, three different neuronal subtypes were identified: peptidergic nociceptor (PEP) cells, non-PEP (NP) cells, and NF (NF) cells.

### Two Step Real-Time qPCR

Two step qPCR analysis was carried out to analyze the relative expression of target genes in B cell-depleted DRG neuron preparations isolated from naïve, alum-injected and alum + KLH-immunized mice, spleens and whole DRG preparations (without B cell depletion) isolated from naïve mice, as well as *in vitro* stimulated B cells and DRG neuron preparations (described above). First, cDNA synthesis was performed using ~1.5 μg of RNA using the ABI High-Capacity cDNA Reverse Transcription Kit (Thermo Fisher Scientific). Second, qPCR reactions were performed (up to 35 cycles) in triplicate using the specific mouse primer pairs and probes (Table 1) (Roche Universal ProbeLibrary), ~25 ng cDNA per well in a 384-well plate using the Eurogentec qPCR MasterMix (AnaSpec, Fremont, CA, USA) and the Roche 480 Light Cycler (Roche Diagnostics). All qPCR samples were run in triplicate. Mouse *Hprt1* was used as the housekeeping gene for normalization. Changes in gene expression, normalized for *Hprt1* expression, were calculated as relative fold-changes using the comparative Ct (ΔΔCt) method (17).

### Statistical Analysis

GraphPad Prism 6.0 software was used for all statistical analysis. Values are presented as mean ± SD. Kruskal–Wallis ANOVA, followed by appropriate *post hoc* tests for multiple comparisons, and Mann-Whitney non-parametric test (for two groups) were performed to determine statistical significance. *P* values equal to or below 0.05 were considered significant.

## Results

### Detection of IgG in Sensory Neurons of Mouse DRG

**Figure 1:**
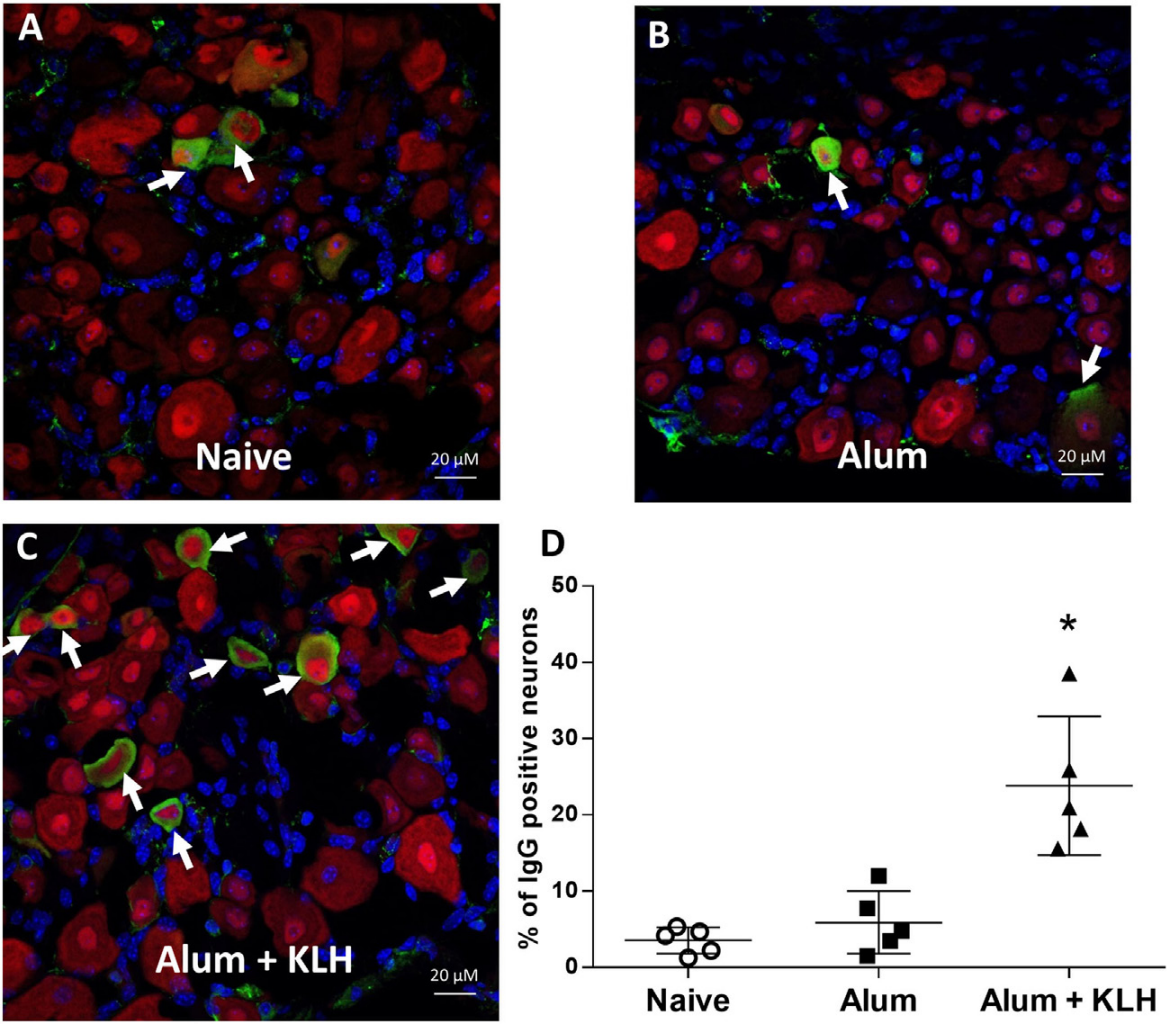
Sensory neurons of the mouse dorsal root ganglia (DRGs) exhibit abundant IgG reactivity. Immunohistochemical staining of whole DRGs isolated from **(A)** naïve, **(B)** alum-injected, and **(C)** alum + KLH-immunized C57BL/6J mice to detect neurons (NeuN, red), IgG (green), and nuclei (DAPI, blue). 400× magnification; scale bar = 20 µm. **(D)** Percentage of double IgG-positive, NeuN-positive neurons (among total NeuN-positive neurons) in DRGs isolated from naïve, alum-injected, and alum + KLH-immunized mice (*n* = 5 per group). A total number of 824, 1,343, and 814 NeuN+ DRG neurons were assessed for immunostaining for the naïve, alum-treated and alum + KLH-immunized mice, respectively. Data are shown as mean percentage ± SD. *P < 0.05 vs naïve DRGs Kruskal–Wallis one way ANOVA (followed by Dunn’s Multiple Comparison Test).

To study the accumulation of IgG in DRG sensory neurons, we performed immunohistochemical analysis of whole mount DRGs collected from naïve (untreated), alum-injected, or alum + KLH-immunized mice using a rabbit F(ab′)_2_ anti-mouse IgG (H+L) antibody. The F(ab′)_2_ fragment against IgG has been used for immunohistochemical staining because it is small and can penetrate tissues/cells, and does not bind to the Fc receptors expressed on the target cells (18, 19). Mice were subjected to either sham immunization, intraperitoneal injection with adjuvant (alum) or immunization with alum + KLH. Animals received an intraperitoneal booster dose on day 14, and whole mount lumbar and thoracic DRGs were isolated from naïve and immunized mice after 4 weeks of immunization. Neuronal populations from DRG sections were stained with labeled anti-NeuN antibodies that recognize the neuronal nuclear protein NeuN. While only sparse IgG-positive staining was observed within the NeuN-positive DRG sensory neurons from the naïve and alum-injected animals, a significant increase in the number of double IgG-positive NeuN-positive neurons was observed in DRGs collected from alum + KLH-immunized mice (Figures 1A–C). Quantitative analysis revealed that IgG-positive staining of the NeuN+ DRG neurons was similar in naïve (3.5% ± 1.8%,) and alum-injected mice (5.9% ± 4.1%) (Figure 1D, a total of 824 and 1,343 NeuN+ neurons were analyzed from naive and alum-injected mice, respectively). By contrast, a significantly higher percentage of NeuN+ DRG neurons stained for IgG following alum + KLH immunization (23.8% ± 9.1%) (Figure 1D, *P* < 0.05, a total of 814 NeuN+ neurons were analyzed). NeuN is most abundant in the neuronal nucleus, and IgG protein is primarily distributed in the cytoplasm of sensory neurons (Figures 1A–C).

### Sensory Neurons From DRG Release Antigen-Specific IgG_1_

**Figure 2:**
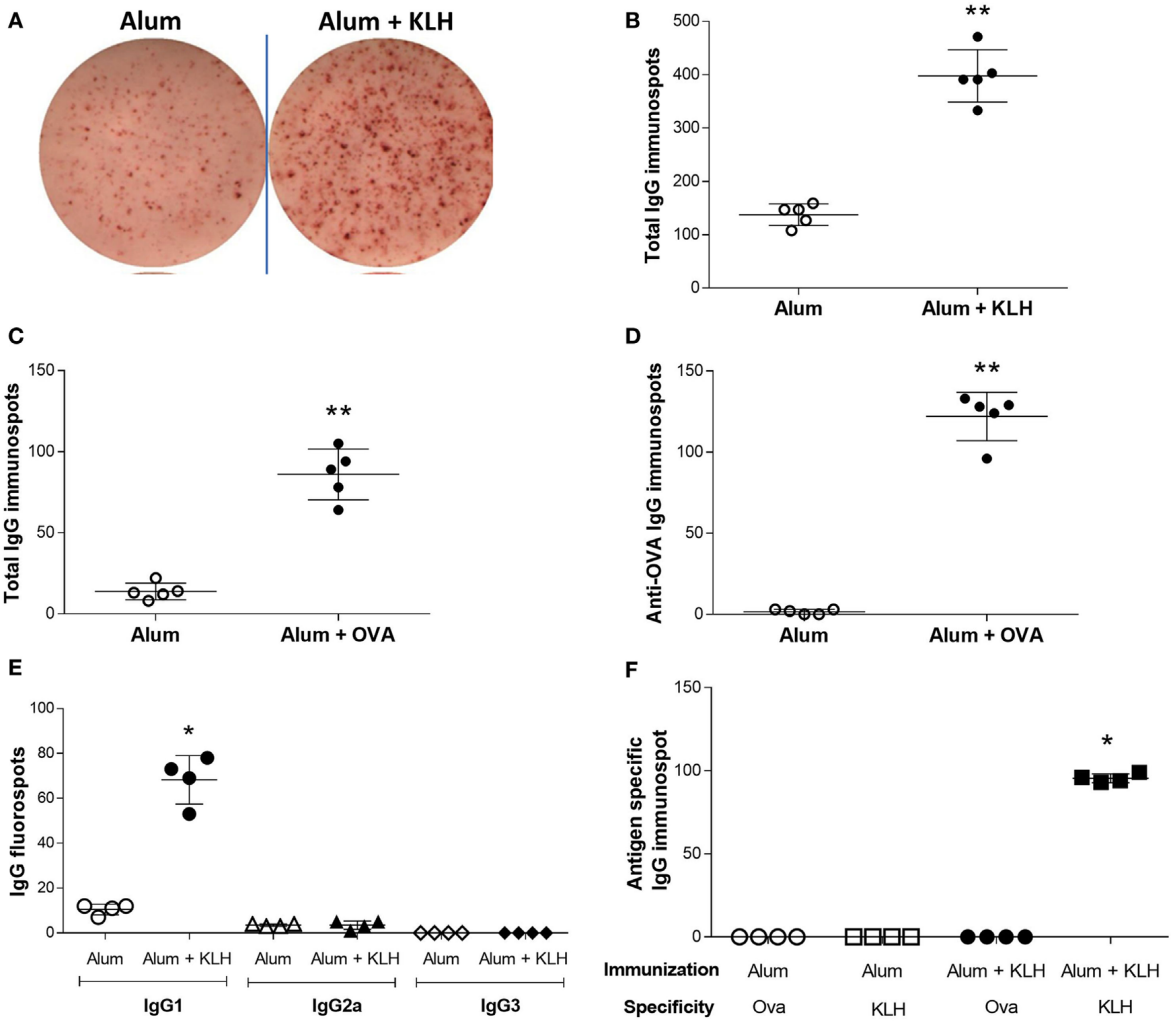
Dorsal root ganglion (DRG) neurons release antigen-specific IgG_1_. **(A)** Representative images from total IgG ELISpot assay performed on DRG neurons isolated from alum-injected and alum + KLH-immunized mice. Quantitation of total IgG-releasing DRG neurons isolated from **(B)** alum-injected and alum + KLH-immunized mice (*n* = 5 per group) or **(C)** alum-injected and alum + ovalbumin (OVA)-immunized mice (*n* = 5 per group) using the ELISpot assay (mean ± SD); **P < 0.01 *vs*. alum-injected (Mann-Whiteny test). **(D)** Quantitation of anti-OVA-releasing DRG neurons isolated from alum-injected and alum + OVA-immunized mice (*n* = 5 per group) using the Anti-OVA IgG ELISpot assay (mean ± SD); **P < 0.01 vs. alum-injected (Mann-Whitney test). **(E)** Quantification of IgG_1_-, IgG_2a_-, and IgG_3_-specific FluoroSpots from DRG neurons isolated from alum-injected and alum + KLH-immunized mice using the IgG isotype FluoroSpot assay; (mean ± SD, *n* = 4 per group); *P < 0.05 *vs*. alum-injected (Mann-Whitney test). **(F)** Quantification of anti-OVA- and anti-KLH-specific IgG immunospots from DRG neurons isolated from alum-injected and alum + KLH-immunized mice (mean ± SD, *n* = 4 per group) using the antigen-specific ELISpot assay; *P < 0.05 *vs*. alum-injected for each antigen (Mann-Whitney test).

To investigate the antigen-specificity of antibodies sequestered in neurons, DRG sensory neurons were cultured on ELISpot plates. A significant increase in the number of total IgG-releasing neurons was observed in DRGs isolated from alum + KLH-immunized mice as compared to alum-injected controls (Figures 2A,B). It is interesting to note that fewer neurons releasing IgG were detected in animals that received only alum (Figures 2A,B), which is in agreement with our immunohistochemical data showing low numbers of sensory neurons sequestering antibodies in DRGs of alum-injected controls (Figure 1B). Similar to alum + KLH-immunization, a significant increase in number of neurons releasing total IgG and OVA-specific IgG was observed in DRGs harvested from alum + OVA-immunized mice (Figures 2C,D). As alum + KLH-immunized mice exhibited robust responses, we utilized this model for our subsequent studies. The majority of these DRG sensory neurons from alum + KLH-immunized mice released IgG_1_ isotype (Figure 2E). To study the antigen specificity of the IgG associated with DRG sensory neurons, we next assessed the number of IgG-releasing neurons in a KLH-specific or an OVA-specific ELISpot assay. A significant increase in KLH-specific neurons was observed in animals immunized with alum + KLH as compared to alum-injected controls (Figure 2F). In contrast, no anti-OVA IgG-releasing neurons were detected in DRGs isolated from either alum-injected or alum + KLH-immunized mice (Figure 2F), indicating that antigen-specific IgG antibodies are associated with DRG sensory neurons in immunized animals.

### DRG Sensory Neurons Lack Expression of Essential Enzymes Required for IgG Synthesis

To assess if sensory neurons from DRGs have the molecular machinery to synthesize antibodies and undergo class-switching, we analyzed the transcriptome of the neuronal population using RNA-seq. DRGs were harvested from animals immunized with alum + KLH (n = 10) or alum-injected controls (n = 10) and RNA-seq analysis was performed using the RNA prepared from pooled DRG neurons for downstream processing to maximize biological diversity. The RNA-seq analysis was primarily used to evaluate the overall transcriptome of the DRG neuron preparations and not to statistically compare the alum vs. alum + KLH-immunized groups. As an exploratory step for characterizing the DRG neuron preparations, RNA-seq was performed using the Illumina NextSeq platform. An average of 17,355 and 17,030 genes were detected (detection > 3 transcripts per million, TPM) in the two groups studied, alum-injected and alum + KLH-immunized, respectively.

Further examination of the RNA-seq data revealed that the expression of neuronal and nociceptor-related gene expression was present in the alum-injected and alum + KLH-immunized groups (Table 2). In contrast, the expression of B lymphocyte, T lymphocyte, mast cell, and basophil-related genes were not observed in either of the groups. Both neuronal preparations show a similar low level of positive regulatory domain zinc finger protein-1 (*Prdm1*) expression (Table 2), which encodes Blimp1, a protein required for the differentiation of B cells into plasma cells (20). These data demonstrate that the enriched neuronal preparation in our study is devoid of B lymphocytes. We next evaluated whether the DRG sensory neuron populations express any of the essential genes required for antibody synthesis and class-switching. No expression of *Rag1, Rag2, Aicda*, or *Ung* genes was observed in either alum-injected or alum + KLH-immunized group (Table 2), indicating that DRG sensory neurons lack the genetic make-up to produce antibodies.

**Table 2:** Expression of neuron, non-neuron, Fc gamma receptor, and antibody recombination gene expression by DRGs.

Neuronal marker genes	RefSeq ID	Protein	Alum-TPM	Alum + KLH -TPM
*Nes*	NM_016701	Nestin	22.40	22.48
*Nefh*	NM_010904	Neurofilament Heavy, NF200	1,020.71	973.48
*Nefl*	NM_010910	Neurofilament Light, NF68	5,259.31	5,048.04
*Nefm*	NM_008691	Neurofilament Medium, NF3	2,262.77	2,181.69
*Rbfox3*	NM_001285438	NeuN	54.34	37.77

**Nociceptor-related genes**				
*Nrp2*	NM_008737	Neuropilin 2	91.98	94.94
*Scn1a*	NM_018733	Na_v_1.1	140.59	161.37
*Scn7a*	NM_009135	Na_v_2.1	693.59	694.48
*Scn10a*	NM_009134	Na_v_1.8	293.78	276.91
*Scn11a*	NM_011887	Na_v_1.9	374.29	330.16
*Trpa1*	NM_177781	TRPA1	153.92	130.88
*Trpv1*	NM_001001445	TRPV1	72.59	73.55
*Trpv2*	NM_011706	TRPV2	10.51	7.61

**B cell marker genes**				
*Cd5*	NM_007650	CD5	0	0
*Cd19*	NM_009844	CD19	0	0
*Cd22*	NM_009845	CD22	0.79	0.76
*Ptprc*	NM_011210	B220; CD45R	0.44	0
*Prdm1*	NM_007548	BLIMP1	3.79	3.07

**T cell marker genes**				
*Cd3e*	NM 007648	CD3e	0.19	0.04
*Cd3g*	NM 009850	CD3y	0	0
*Cd8a*	NM_001081110	CD8A	0	0
*Cd80*	NM_009855	CD80	0.89	0.93

**Basophil and mast cell genes**				
*Cd200r3*	NM_029018	CD200R3	0	0
*Cd200r3*	NM_001128132	CD200R3	0	0
*Cd200r3*	NM_027578	CD200R3	0	0
*Anpep*	NM_008486	CD13	0.13	0.16
*Il3ra*	NM_008369	IL-3Ra	0.19	0.04

**Fc gamma receptor genes**				
*Fcgr1*	NM_010186	FcyR1	0.07	0.04
*Fcgr2b*	NM 010187	FcyR2b	5.67	4.44
*Fcgr2b*	NM 001077189	FcyR2b	0	0.66
*Fcgr3*	NM_010188	FcyR3	0.3	0.3
*Fcgr4*	NM_144559	FcyR4	4.32	3.97
*Fcgrt*	NM_010189	FcRn	2.31	1.40

**Ab recombination genes**				
*Aicda*	NM_009645	AID	0.05	0.02
*Rag1*	NM_009019	RAG1	0.08	0.06
*Rag2*	NM_009020	RAG2	0.61	0.56
*Ung*	NM_011677	Uracil-DNA glycosylase (UNG)	1.27	0.71

*For RNA-Seq: RNA was isolated from DRG neurons collected from alum-injected mice and alum + KLH-immunized mice (*n* = 10 animals per group – pooled). The values expressed are TPM values for each pooled sample (without SDs). TPM, transcripts per million (relative abundance of gene expression based on the length of each gene and for sequencing depth)*.

**Figure 3:**
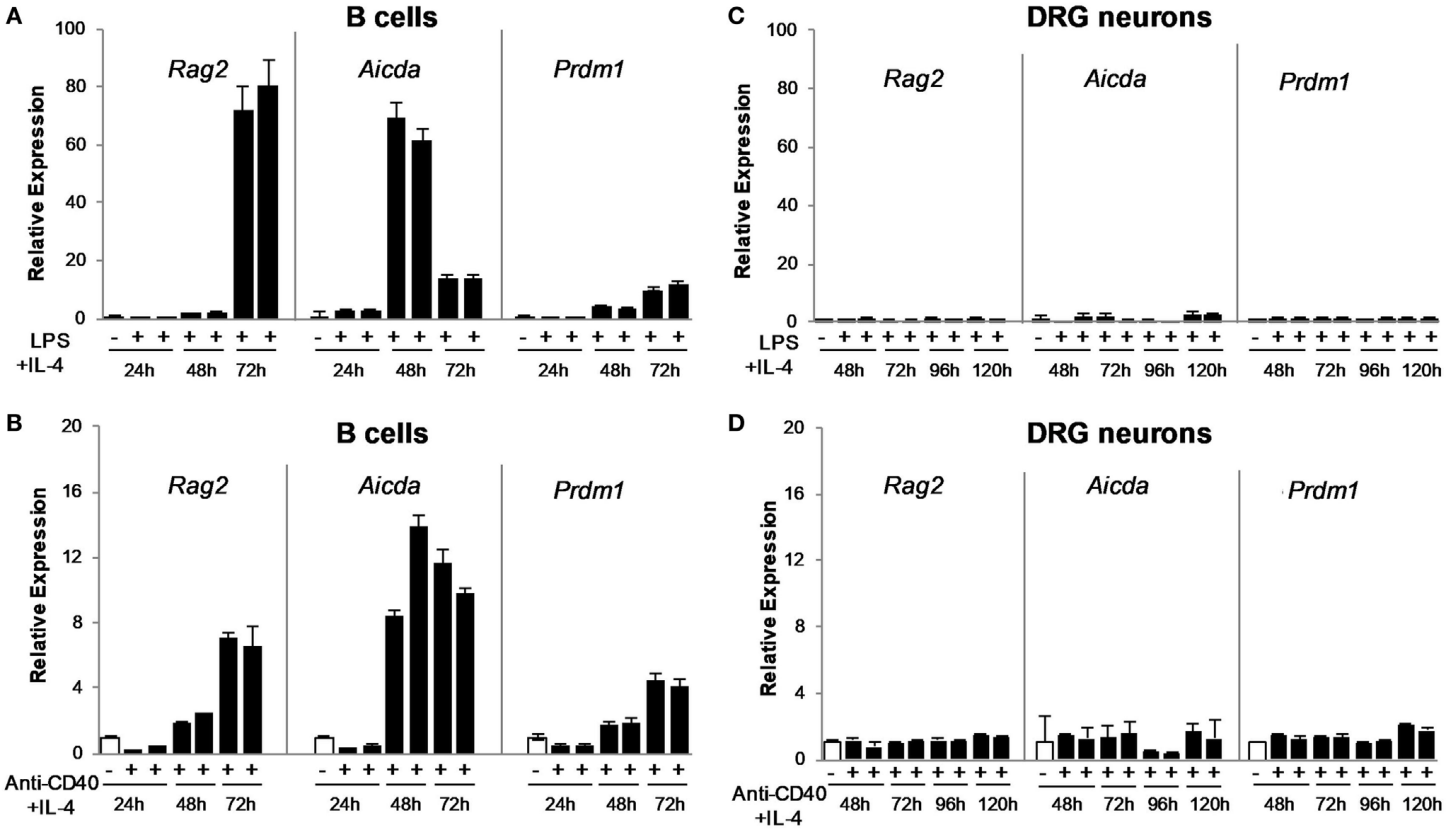
*In vitro* stimulated splenic B cells, but not dorsal root ganglion (DRG) sensory neurons, express B cell activation markers associated with antibody synthesis. Naïve splenic B cells stimulated *in vitro* for 24–72 h with LPS + IL-4 **(A)** or anti-CD40 + IL-4 **(B)** express genes associated with antibody production (*Rag2* and *Aicda*), as well as *Prdm1*, a marker of activated B cells/plasma cells. Stimulated B cells are compared to naïve freshly isolated, untreated B cells. In contrast, DRG neurons stimulated *in vitro* with LPS + IL-4 **(C)** and anti-CD40 + IL-4 **(D)** for up to 120 h do not exhibit gene expression associated with antibody production (*Rag2* and *Aicda*) or *Prdm1* when compared to untreated DRG neurons. Stimulated DRG neurons are compared to vehicle-treated cells at each time point. Data are shown as mean ± SD quantitative PCR (qPCR) data for duplicate samples.

To confirm the RNA-seq data, we validated the expression of genes required for antibody synthesis and B cell differentiation into plasmablasts/plasma cells at different time points post-immunization. DRG neuronal preparations were isolated from animals (alum-injected controls or alum + KLH-immunized) at three different time points (*n* = 10 mice per group): post-primary immunization, post-first booster and post-second booster as described in the Section “Materials and Methods.” The expression pattern of four genes related to antibody synthesis (*Rag1, Rag2, Aicda*, and *Ung*) and two genes corresponding to B lymphocyte/plasma cell markers (*Cd19* and *Prdm1*) were validated by quantitative real-time RT-PCR. As expected, high levels of the B cell marker, *Cd19*, and low-moderate levels of *Rag1, Rag2, Aicda*, and *Ung* were observed in the naïve spleen. However, DRG sensory neurons failed to express *Rag1, Aicda* or *Ung* at any of the time points studied (Table 3). Our preliminary RNA-seq data confirmed the expression of *Il4ra* (the gene that encodes the alpha chain of the IL-4 receptor, a type I transmembrane protein that can bind IL-4 and IL-13 to regulate IgG production) in DRG sensory neurons (TPM value ~5) and the expression of the *Tlr4* gene (that encodes the receptor for LPS) (TPM value ~7). To address the possibility that the molecular events related to antibody synthesis are not operative during the DRG sample collection and analysis, but may be induced in a similar manner as activated B cells, we stimulated DRG sensory neurons (or splenic B cells as a positive control) *in vitro* and assessed the expression of *Rag2* and *Aicda* at three different time points post-stimulation. We used two conditions for *in vitro* activation of DRG sensory neurons and B cells: LPS + IL-4 (T-independent) or anti-CD40 + IL-4 (T-dependent) and no treatment (vehicle). As reported previously (21, 22), stimulation of mature mouse B cells with LPS + IL-4 resulted in the expression of both *Rag2* and *Aicda in vitro* in a time-dependent manner (Figure 3). In contrast, activation of DRG sensory neurons with either T-independent (LPS + IL-4) or T-dependent (anti-CD40 + IL-4) stimuli failed to induce either *Rag2* or *Aicda* gene expression (Figure 3). These data confirm that DRG sensory neurons lack the expression of essential enzymes required for IgG synthesis.

**Table 3:** *In vivo* analysis of gene expression by Q-PCR.

	Day 0		Day 14		Day 28			
					
	Alum	Alum + KLH	Alum	Alum + KLH	Alum	Alum + KLH	Naïve DRG	Naïve Spleen
*Rag1*	1.0 ± 0.2	2.0 ± 0.2	0.6 ± 0.4	0.6 ± 0.3	1.2 ± 0.4	0.7 ± 0.2	0.7 ± 0.2	41.05 ± 5.3
*Rag2*	1.0 ± 0.4	1.3 ± 0.6	12.2 ± 3.1	57.4 ± 9.9	18.0 ± 1.6	43.4 ± 14.7	1.8 ± 0.4	2,083 ± 370
*Aicda*	1.0 ± 0.0	0.0 ± 0.0	0.0 ± 0.0	0.0 ± 0.0	0.3 ± 0.0	0.4 ± 0.0	0.0 ± 0.0	750 ± 149
*Ung*	1.0 ± 0.1	1.5 ± 0.2	0.7 ± 0.2	1.1 ± 0.2	1.4 ± 0.1	1.3 ± 0.3	0.8 ± 0.1	21.7 ± 1.3
*CD19*	1.0 ± 0.0	0.4 ± 0.2	0.8 ± 0.0	1.2 ± 0.0	1.8 ± 0.4	1.6 ± 1.7	8.9 ± 2.5	5,7300 ± 8,261
*Prdm1*	1.0 ± 0.1	1.1 ± 0.2	0.3 ± 0.0	0.4 ± 0.0	0.9 ± 0.2	0.7 ± 0.2	0.3 ± 0.0	14.7 ± 1.8

*Mice (n = 3 per group) were injected with either alum alone or alum + KLH on days 0, 14, and 28; DRG neurons were isolated 24 h after the last injection as described in the Section “Materials and Methods.” RNA was isolated from resultant DRG neurons, as well as from DRG neurons and spleens harvested from naïve mice. Gene expression was analyzed by qPCR. Data are shown as relative fold changes (mean ± SD) normalized to alum-injected group. Results reveal the lack of *Rag1, Aicda*, and *Ung* in DRG neurons across time points and the presence of gene expression in the Naïve spleen*.

### Transgenic *Ighg1*-tdTomato Mice Do Not Express tdTomato in DRG Sensory Neurons

**Figure 4:**
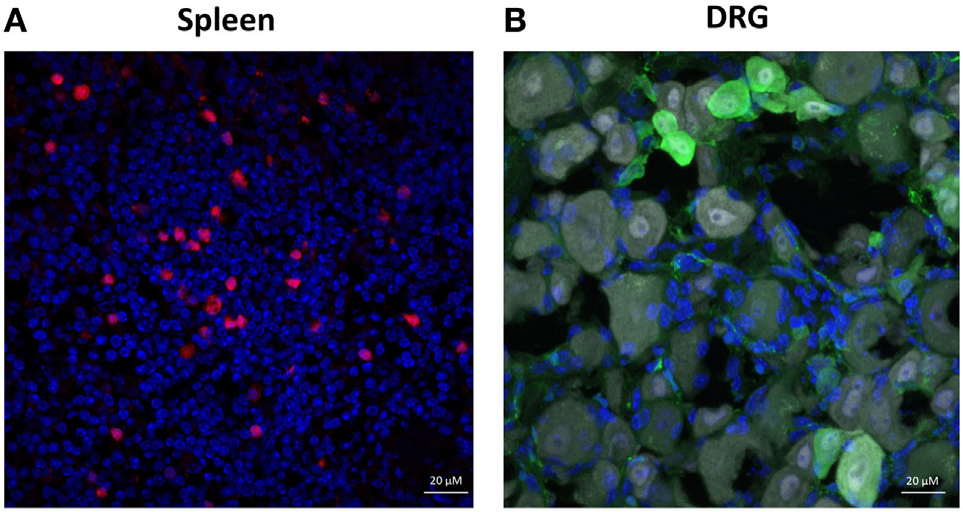
Mouse dorsal root ganglion (DRG) neurons lack Igγ1 chain expression. Immunohistochemical staining of spleen and whole DRGs isolated from alum + KLH-immunized mice expressing tdTomato under the control of the *Ighg1* promoter. **(A)** Spleens express Igγ1 chain (red) (DAPI = blue). **(B)** DRG neurons express NeuN (gray) and IgG (green), but not Igγ1 chain (red; tdTomato) (DAPI = blue). 400× magnification; scale bar = 20 µm.

Casola et al (23) have produced a transgenic *Ighg1*-cre mouse that can be used in combination with a reporter gene to analyze the transcription of Igγ1 constant region gene segment (Cγ1) in antibody expressing cells. We utilized the *Ighg1*-cre mice to induce expression of a fluorescent reporter protein, tdTomato, in antibody-expressing cells because IgG_1_ was found to be the most prominent subtype in the DRG neurons (Figure 2E). The elimination of the STOP cassette and subsequent tdTomato expression was achieved by crossing *Ighg1*-cre mice with B6.Cg-*Gt(ROSA)26Sor^*tm14(CAG-tdTomato)Hze*^*/J mice, resulting in tdTomato expression mostly in cells expressing Cγ1 and hence, IgG_1_. Transgenic mice (carrying both cre and tdTomato) were indistinguishable from their WT littermate controls (carrying only cre or tdTomato) in terms of developmental features. tdTomato is one of the brightest among the red-fluorescent proteins and well-suited for immunohistochemical analysis. Accordingly, we found that the tdTomato reporter mice had increased numbers of tdTomato + cells in their spleens following immunization (Figure 4A). However, no colocalization of tdTomato fluorescent signal and antibody staining was observed in sensory neurons following immunization (Figure 4B). As tdTomato expression is driven by the transcription of Cγ1 (of IgG_1_), these data confirm that *Ighg1* are not expressed in sensory neurons from DRG.

### DRG Sensory Neurons Lacking Neuronal *Rag2* Expression Contain Antigen-Specific Antibodies

**Figure 5:**
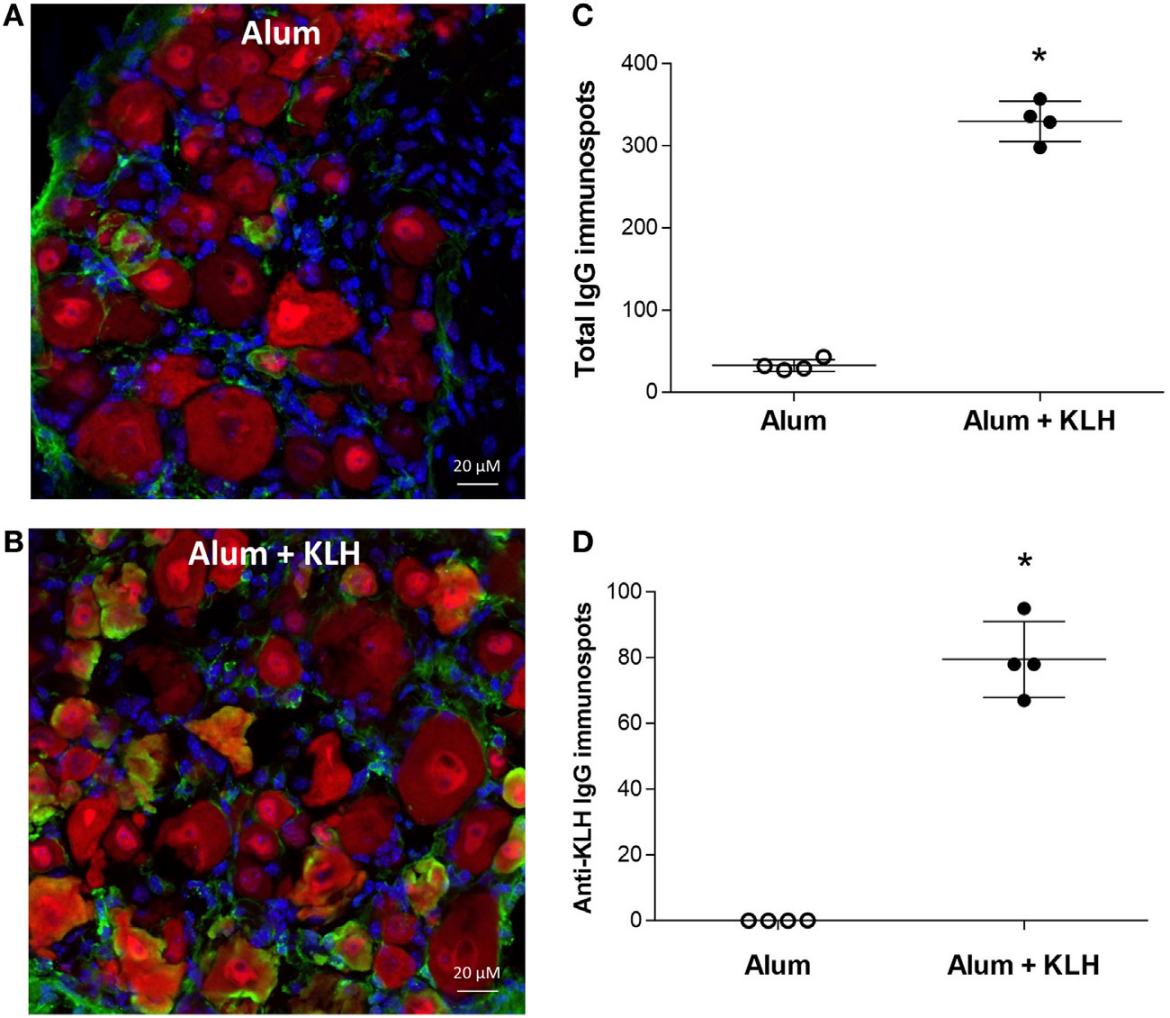
Dorsal root ganglion (DRG) neurons from mice lacking neuronal-specific *Rag2* expression contain antigen-specific antibodies. Immunohistochemical staining of whole DRGs isolated from alum-injected **(A)** and alum + KLH-immunized **(B)** neuronal–specific *Rag2* knock-out (KO) mice for IgG (green), neurons (NeuN, red), and nuclei (DAPI, blue). 400× magnification; scale bar = 20 µm. **(C)** Quantitation of IgG-releasing DRG neurons isolated from alum-injected and alum + KLH-immunized neuronal-specific *Rag2* KO mice using the ELISpot assay (mean ± SD, *n* = 4 per group); *P < 0.05 *vs*. alum-injected (Mann-Whitney test). **(D)** Quantification of anti-KLH-specific IgG immunospots from DRG neurons isolated from alum-injected and alum + KLH-immunized neuronal-specific *Rag2* KO mice (mean ± SD, *n* = 4 per group) using the antigen-specific ELISpot assay *p < 0.05 *vs*. alum-injected (Mann-Whitney test).

Because neuronal *Rag2* expression has been implicated in previous work (24–27), here we crossed floxed-*Rag2* transgenic mice with synapsin-cre transgenic mice generating null mutants that selectively lack *Rag2* expression in neurons (with abundant *Rag2* expression in B cells). Analysis of immunoglobulin reactivity in the DRGs of null mutants revealed the presence of IgG-positive NeuN-positive neurons in both alum-injected (Figure 5A) and alum + KLH-immunized (Figure 5B) mice. Similar to previous observations with WT mice (Figures 2A,B,F), significantly higher numbers of IgG-releasing and anti-KLH IgG-releasing DRG neurons were observed in the alum + KLH group when compared to the alum-injected group even in the absence of *Rag2* expression (Figures 5C,D). Taken together, our results indicate that sensory neurons from DRGs lack the enzymes and cannot synthesize antibodies.

### Antibodies Found in DRG Neurons Are Released by B Cells

**Figure 6:**
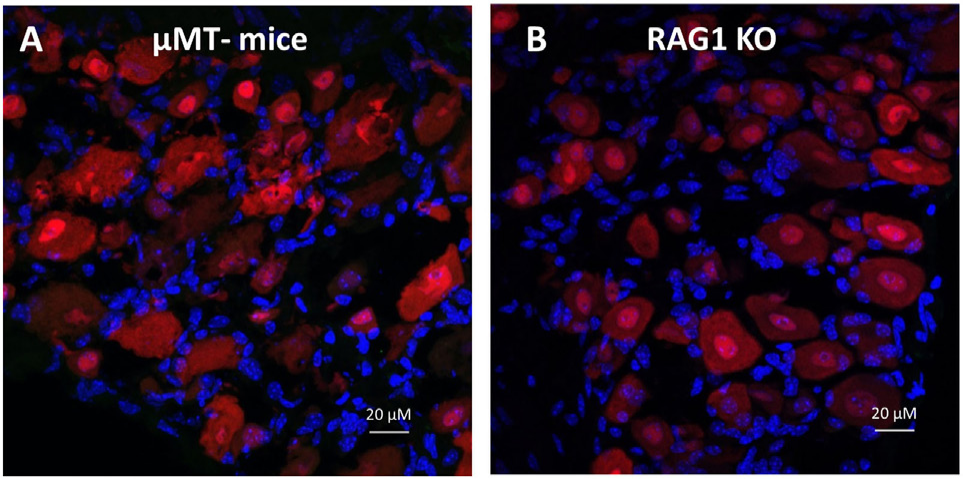
Dorsal root ganglion (DRG) neurons from mice deficient in B cell-derived IgGs [μMt and *Rag1* knock out (KO)] lack antigen-specific IgG immunoreactivity. Immunohistochemical staining of whole DRGs isolated from alum + KLH-immunized μMt **(A)** and *Rag1* KO **(B)** mice stained for neurons (NeuN, red), IgG (green), and nuclei (DAPI, blue), 400× magnification; scale bar = 20 µm.

**Figure 7:**
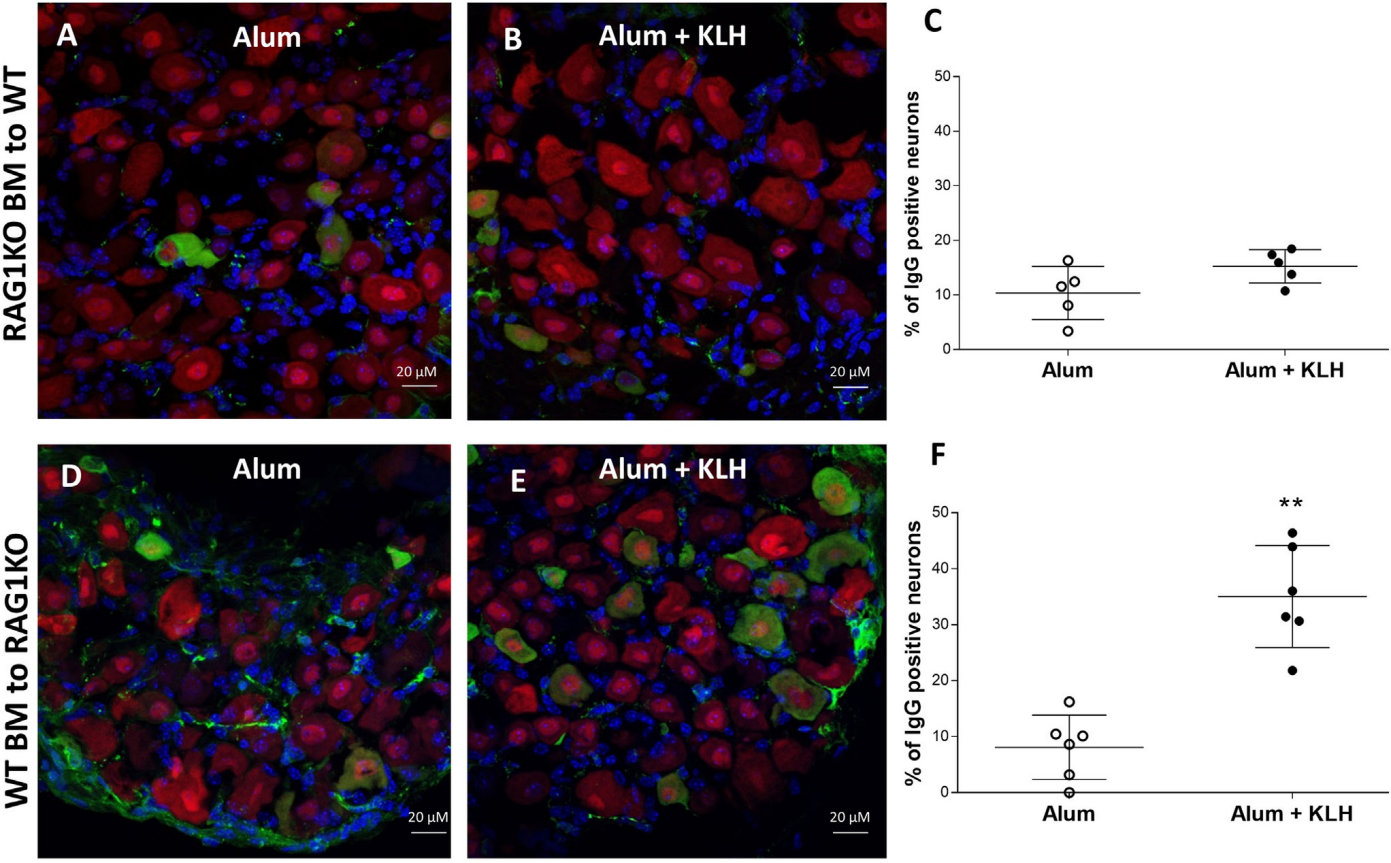
Dorsal root ganglion (DRG) neurons from chimeric mice with *Rag1* knock out (KO) BM lack immunization-induced antibody accumulation. Immunostaining of whole DRGs isolated from alum-injected **(A)** and alum + KLH-immunized **(B)** chimeric mice (*Rag1* KO BM to WT) for neurons (NeuN, red), IgG (green), and nuclei (DAPI, blue). 400× magnification; scale bar = 20 µm. **(C)** Percentage of double IgG-positive, NeuN-positive neurons (among total NeuN-positive neurons) in DRGs from isolated from alum-injected and alum + KLH-immunized chimeric mice (*Rag1* KO BM into WT mice); data are shown as mean percentage ± SD; (*n* = 5 per group). Immunostaining of whole DRGs isolated from alum-injected **(D)** and alum + KLH-immunized **(E)** chimeric mice (WT BM into *Rag1* KO) for neurons (NeuN, red), IgG (green), and nuclei (DAPI, blue). 400× magnification; scale bar = 20 µm. **(F)** Percentage of double IgG-positive, NeuN-positive neurons (among total NeuN-positive neurons) in DRGs isolated from alum-injected and alum + KLH-immunized chimeric mice (WT BM to *Rag1* KO); (mean percentage ± SD, *n* = 6 per group). **P < 0.05 *vs*. alum-injected (Mann-Whitney test).

Accordingly, we reasoned that the neurons from DRGs sequester and retain antibodies produced by plasma cells, and therefore, DRGs isolated from mice incapable of producing B cell-derived antibodies would contain no antigen-specific antibodies. To address this question, we utilized mice with a homozygous targeted disruption of the membrane exon of the Ig mu-chain (μMT mice) that are deficient of mature peripheral B cells as well as *Rag1* KO mice that do not produce mature T and B lymphocytes (28–30). Immunohistochemical analysis revealed that DRGs from either μMT or *Rag1* KO mice do not contain antigen-specific antibodies (Figures 6A,B). Next, we generated chimeric mice by transferring *Rag1* KO BM to WT mice or WT BM to *Rag1* KO mice. After confirming the reconstitution of the recipient animals (data not shown), the chimeric mice received alum injections or alum + KLH immunizations as described. Immunohistochemical analysis of chimeric mice with *Rag1*-deficient BM (*Rag1*-negative hematopoietic cells, but *Rag1*-positive DRG sensory neurons) revealed that in the absence of antibody secreting plasma cells, no IgG reactivity is observed in sensory neurons from DRGs isolated from either alum-injected (Figure 7A) or alum + KLH-immunized groups (Figures 7B,C). In contrast, IgG-positive neuronal populations were observed in DRGs from alum-injected chimeric mice with WT BM (*Rag1*-positive hematopoietic cells, *Rag1*-negative sensory neurons) (Figure 7D). Furthermore, immunization with alum + KLH significantly increased the number of IgG-positive sensory neurons in the DRGs as observed with WT mice (Figures 7E,F).

**Figure 8:**
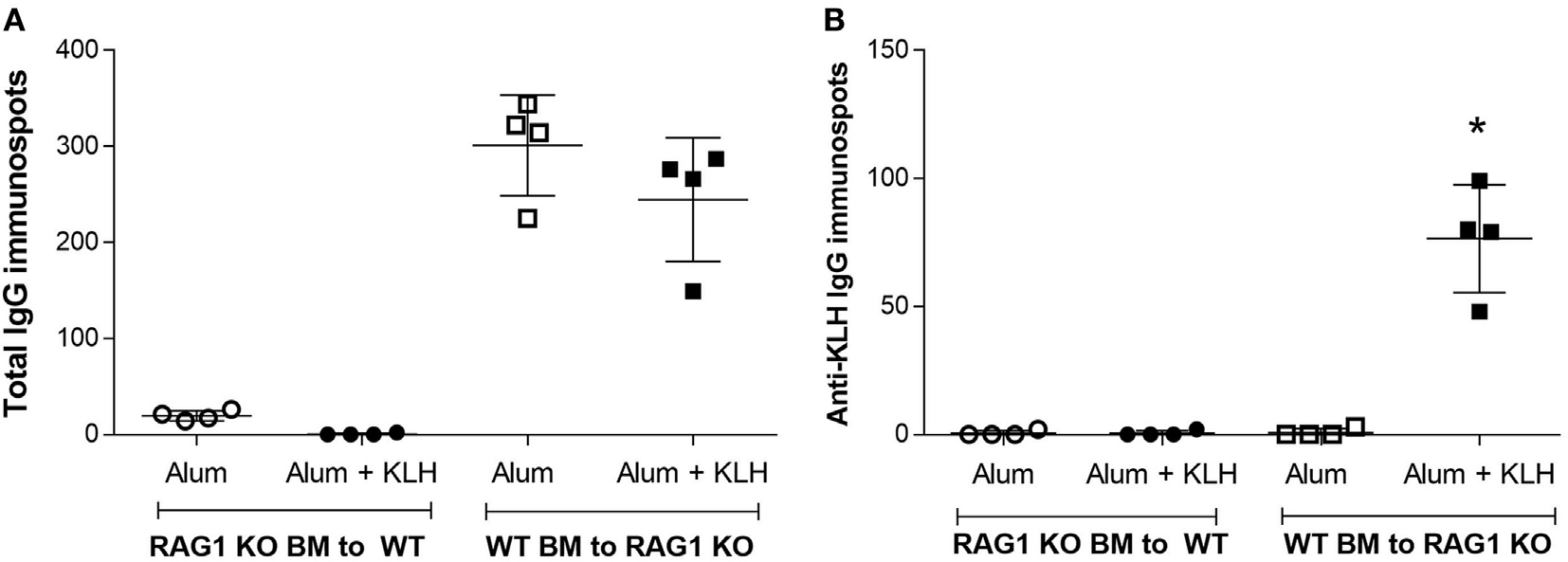
Dorsal root ganglion (DRG) sensory neurons from mice with *Rag1*-positive hematopoietic cells, but not from mice with *Rag1*-negative hematopoietic cells, release antigen-specific IgG. Detection of IgG-releasing cells using the ELISpot assay. Quantification of **(A)** total IgG and **(B)** anti-KLH-specific IgG immunospots produced by DRG neurons isolated from alum-injected and alum + KLH-immunized chimeric mice [*Rag1* knock-out (KO) bone marrow (BM) into wild-type (WT) and WT BM into *Rag1* KO]. (Mean ± SD, *n* = 4 per group). *P < 0.05 *vs*. alum-injected (Mann-Whitney test).

We next analyzed the immunoglobulin release by DRG sensory neurons from chimeric mice using both total IgG and KLH-specific IgG ELISpot assays. Increased IgG-positive neuronal cells are observed in the DRGs harvested from chimeric mice with WT BM, but not in the chimeric mice with *Rag1*-deficient BM (Figure 8A). Analysis of KLH-specific IgG-releasing cells revealed a significant increase in anti-KLH IgG-positive DRG sensory neurons in the DRGs isolated from alum + KLH-immunized chimeric mice with WT BM (Figure 8B). In contrast, no anti-KLH IgG-secreting DRG sensory neurons are observed in immunized chimeric mice with *Rag1*-deficient BM (Figure 8B). Administration of alum alone also increased the number of total IgG-releasing DRG sensory neurons, but not anti-KLH IgG-positive neurons (Figures 8A,B). Together, these data suggest that DRG sensory neurons not only sequester and retain antibodies secreted by plasma B cells but also can release these antibodies.

## Discussion

Here we demonstrate that antigen-specific antibodies are sequestered in mouse DRG sensory neurons. A significantly higher number of DRG sensory neurons from immunized animals showed sequestration of antigen-specific antibodies as compared to alum-injected control or naïve mice. RNA-seq analysis and qPCR of the neuronal transcripts revealed that sensory neurons from DRGs lack the genetic machinery to produce antibodies. Using transgenic and chimeric animals, we further confirmed that DRG sensory neurons do not synthesize antigen-specific antibodies, but sequester the antibodies released by plasma cells.

As shown in Figure 1, DRG neurons isolated from naive mice and alum-injected mice had similar low percentages of IgG-containing NeuN+ neurons (~4–6%), while DRG neurons isolated from mice immunized with alum + KLH showed significantly higher percentages (~24%). Antibody localization appears intracellular. However, the subcellular localization of IgG within NeuN+ DRG neurons remains to be determined. All additional experiments included the alum-injected and alum + KLH-immunized groups. Although this study design limited our ability to compare DRG neurons obtained from naive *vs*. alum-treated mice, it focused our studies on assessing antigen-specific antibody responses in alum-injected vs. alum + KLH-immunized mice. The anti-KLH antibodies released by DRG neurons were primarily IgG1 (not IgG2 or IgG3) (Figure 2D). We did not assay for IgA, IgE, or IgM antibodies. Future studies will examine what stimuli (e.g., antigen, capsaicin, or KCl) trigger IgG release by DRG neurons.

Antibodies are normally synthesized by mature plasma cells. However, recent accumulating evidence suggests that non-lymphoid cells, such as human sperm (31), hepatocytes (32), certain carcinoma cells (25, 32, 33), and neurons from the central nervous system (34–37), also express genes required for antibody synthesis and produce antibodies. Here, we demonstrate that although DRG sensory neurons exhibit localization of antigen-specific antibodies, the neuronal cells do not express the essential enzymes required for generating antibody diversity. V(D)J recombination is tightly regulated during B cell differentiation by transcriptional regulation of lymphoid-specific recombinase genes encoding RAG1 and RAG2 enzymes (38–41). In mature B cells, antigenic stimulation induces somatic hypermutation, gene conversion, and class switch recombination leading to generation of a secondary repertoire of high-affinity antigen-specific antibodies (42, 43). Activation-induced cytidine deaminase (AID), an enzyme encoded by the *Aicda* gene, is responsible for regulating and coordinating these three genetic rearrangements in mature B cells (42, 44). AID generates antibody diversity by converting cytosine to uracil within the immunoglobulin loci resulting in mismatch mutations. The uracil-guanine mismatch created by AID is repaired by uracil-DNA glycosylase (UNG), a DNA-repair enzyme encoded by the gene *Ung*, introducing base-pair changes resulting in antibody diversity (42). The DRG neuronal transcriptome analysis here revealed the absence of *Rag1, Rag2, Aicda*, and *Ung* transcripts. Furthermore, *in vitro* activation of DRG sensory neurons with LPS and IL-4 (to induce T-independent class switch recombination) or anti-CD40 and IL-4 (to induce T-dependent class switch recombination) did not induce significant increases in expression of either *Rag1, Rag2, Aicda*, or *Ung* genes. Moreover, a comprehensive transcriptome analysis of 622 hand-picked single neurons harvested from lumbar DRGs of naïve mice showed no detectable *Rag1, Rag2*, or *Aicda* expression (16).

An interesting finding in the current study is the marked discordance in the expression of *Rag1* and *Rag2* transcripts in DRG sensory neurons harvested from immunized mice. Amplification of the neuronal transcripts showed low levels of *Rag2* but no *Rag1* transcripts present in DRG sensory neurons. It is unlikely that DNA contamination contributed to the results, as all samples were treated with DNase before analysis, and intron spanning primers were used. These data rather suggest that *Rag1* and *Rag2* are differentially expressed in immunized mice. As discussed earlier, expression of *Rag1* and *Rag2* is tightly controlled and occurs primarily during the early developmental stages of T and B cells. A *cis*-regulatory element located in the RAG locus regulates coordinated expression of *Rag1* and *Rag2*. However, a less-strict control of transcription within the RAG locus results in functionless expression of *Rag2* transcript in the brain and other non-lymphoid tissues (27). Although expression of *Rag1* and *Rag2* transcripts have been reported in a variety of non-lymphoid cells, including different cancer cell types, epithelial cells and neurons (34, 45, 46), to our knowledge, no functional role for these enzymes have been identified in non-lymphoid cells.

In order to investigate the functional role of the RAG2 enzyme for antibody synthesis in neurons, we developed a neuron-specific *Rag2* KO mouse to detect if antibodies are sequestered in neurons in the absence of neuronal *Rag2* gene expression. We immunized the neuronal *Rag2* KO mice with alum + KLH and observed a significant increase in antibody sequestration, total IgG and anti-KLH IgG-releasing neurons in DRGs from immunized mice even in the absence of *Rag2* expression in neurons. Mice deficient in either RAG1 or RAG2 enzymes lack mature lymphocytes and fail to generate antibodies due to their inability to initiate V(D)J rearrangement (30, 47). In the absence of V(D)J recombination and mature B cells, *Rag1* KO mice completely lacked antibody localization in their DRG sensory neurons. Consistent with this observation, no localization of antibodies was observed in mice homozygous for an inactivating mutation of the membrane exon of their immunoglobulin μ chain gene (μMT) that results in mature B cell deficiency and no antibody production (29). Adoptive transfer of WT BM into *Rag1*-deficient mice restored antibody sequestration in DRGs. Together, these results indicate that DRG sensory neurons sequester antibodies released by mature B cells.

Antibody accumulation has been described in brain tissues and cerebrospinal fluid in both healthy and pathological conditions (35, 37, 48, 49). Antigen-antibody immune complexes directly activate sensory neurons resulting in an increase in intracellular calcium (9, 50), initiation of action potentials (8, 50), and the release of neurotransmitters from DRG neurons (9). The presence of antibodies in the nervous system could be attributed to receptor-mediated uptake of antibodies by neurons. Immunoglobulin G and its receptors have been observed in the human nervous system (34), rodent brains (9, 35, 37, 51), and zebrafish sensory neurons (24, 46). Expression of different subtypes of Fcγ receptors have been shown in Purkinje cells, parvalbumin neurons, primary neuronal cultures, and sensory neurons (52–54). While RNA-seq and qPCR (data not shown) failed to detect the expression of either *Fcgr1* or *Fcgr3* in DRG sensory neurons, the expression of one *Fcgr2b* variant and *Fcgrt* were detected (Table 2). Consistent with our data, single cell analysis of the DRG neuron transcriptome revealed a lack of expression of Fcγ receptors 1 and 3 (16). However, the presence of FcRn, an MHC class I related antibody receptor, has been detected in sensory neurons (16). FcRn, a product of the *Fcgrt* gene, plays a pivotal role in passively transferring IgG within and across multiple cell types (55–59) and protecting IgG from degradation (59–61). Mice genetically deficient in *Fcgrt* (lacking FcRn) have significantly reduced plasma half-life of IgG and exhibit hypogammaglobulinemia (60–62). In the central nervous system, FcRn expressed by endothelial cells has been implicated in antibody transport across the blood brain barrier (63). Similar to the situation in the central nervous system, it is possible that in DRGs, FcRn mediates the transport of antibodies into sensory neurons and protects the IgGs from degradation within the neuronal cells. Further studies are required to address the functions of Fcγ receptors and FcRn in peripheral DRG sensory neurons. Future studies using *in vitro* and *in vivo* methods including single cell qPCR (to compare neurons that sequester IgG *vs*. those that do not) will provide insight on how and why antigen-specific antibodies are taken up by DRG neurons.

Several possibilities exist that may explain the accumulation of antibodies in sensory neurons. Antibody uptake by neurons may have a self-protecting effect. Using a murine stroke model, Arumugam et al have demonstrated that intravenously injected IgG protects the brain against neuronal death by neutralizing the effects of complement (64). In a mouse model of Alzheimer’s disease, antibodies assisted clearance of amyloid β-peptide from the CNS into the circulation *via* FcRn (65). Antigen-specific antibodies also aid in clearance of neuronal-specific pathogens from neurons. Treatment of encephalitis virus-infected primary cultured neurons with virus-specific antibodies clears the infectious encephalitis virus from neurons by restricting viral gene expression (66). Accordingly, immunodeficient *scid* mice infected with the encephalitis virus develop persistent non-lethal central nervous system infection (66). It is also possible that invading pathogens get opsonized by the locally released antibodies and are subsequently phagocytosed by glial cells (67). In addition, antigen-antibody complexes may mediate hyperalgesia by activating peripheral sensory neurons. Numerous antigen-specific immune-related disorders are often accompanied by pain. These disorders include autoimmune diseases such as rheumatoid arthritis (68) and Guillain-Barre syndrome (69), viral reactivation syndromes such as herpes zoster (70, 71), and allergic diseases such as atopic and allergic contact dermatitis (72, 73). Elevated levels of antigen-specific antibodies, especially IgG, and immune complexes are found in the serum and affected tissues in these diseases. Immune complexes directly activate nociceptive DRG neurons in a TRPC3-receptor dependent manner, and increase neuronal excitability and thus, potentially contribute to pain (7, 8). Recent studies have demonstrated that intradermal injection of immune complexes dose-dependently produces mechanical and thermal hyperalgesia in the hind paws of rats that can be alleviated by localized administration of non-specific antibody (74). Finally, it is important to note that our studies were confined to DRG sensory neurons. We did not study other sensory neurons (e.g., nodose or trigeminal ganglia).

Our studies demonstrate that peripheral DRG sensory neurons sequester antibodies made by plasma cells. In concluding that DRG neurons do not produce antibodies we are at risk of a type II error (in failing to reject a null hypothesis that is actually false). We have attempted to reduce the risk of this error by analyzing large numbers of DRG neurons from the mice and by testing the same hypothesis using multiple approaches and models [qPCR, RNA-seq, immunostaining, and both *in vitro* and *in vivo* models (using multiple genetically modified mice and BM transfer techniques)]. Therefore, we are confident that our results demonstrate that DRG neurons sequester antigen-specific antibodies. These antigen-specific antibodies may play an important role in protecting DRG neurons from pathogen-induced damage, or in inducing antigen-specific hyperalgesia. These findings, together with the previous studies, implicate immune complexes in mediating neuro-immune crosstalk between DRG sensory neurons and antibodies in both physiological and pathological conditions.

## Ethics Statement

All animal experiments were performed in accordance with the National Institutes of Health Guidelines under protocols approved by the Institutional Animal Care and Use Committee (IACUC) and the Institutional Biosafety Committee (IBC) of the Feinstein Institute for Medical Research, Northwell Health, Manhasset, NY, USA.

## Author Contributions

MKG, CNM, KJT, and SSC designed research; MKG, PKC, AS, GHI, MA, GK, AL, CNM and SSC performed research; MKG, PKC, AS, GK, JFG, DM, JA, UA, TRC, CNM, KJT, and SSC analyzed and interpreted data; MKG, TRC, CNM, KJT and SSC wrote the article; MM, CP, MAC, TWM, CB, BS and BD provided additional comments and contributed to finalizing the article.

## Conflict of Interest Statement

The authors declare that the research was conducted in the absence of any commercial or financial relationships that could be construed as a potential conflict of interest.

## Footnotes

^1^http://www.nature.com/nbt/journal/v34/n5/full/nbt.3519.html (Accessed: December 29, 2017).

^2^https://www.ncbi.nlm.nih.gov/geo/query/acc.cgi?acc=GSE108428 (Accessed: December 29, 2017).

## Supplementary Material

Figure S1 Cellular composition of DRG neuron preparations. The principal DRG cell types in the sensory neuron preparations isolated from DRGs collected from alum-injected and alum + KLH-immunized mice include neuronal cells: peptidergic nociceptor (PEP) cells, non-peptidergic nociceptor (NP) cells, and neurofilament containing (NF) cells, and non-neuronal (NoN) cells. The cell types were defined by Usoskin and colleagues (16), and the relative proportions were computed using RNA-seq deconvolution analyses, as described in the Section “Materials and Methods.”
